# Wearable Devices for Caloric Intake Assessment: State of Art and Future Developments

**DOI:** 10.2174/1874434601711010232

**Published:** 2017-10-31

**Authors:** Maria Laura Magrini, Clara Minto, Francesca Lazzarini, Matteo Martinato, Dario Gregori

**Affiliations:** 1 Unit of Biostatistics, Epidemiology and Public Health, Department of Cardiac, Thoracic and Vascular Sciences, University of Padova, Padova, Italy; 2 Department of Gastroenterology, University of Padova, Padova, Italy

**Keywords:** Wearable devices, Caloric intake monitoring, Non-communicable diseases, Body Mass Index, Bite, Armband

## Abstract

**Background:**

The self-monitoring of caloric intake is becoming necessary as the number of pathologies related to eating increases. New wearable devices may help people to automatically record energy assumed in their meals.

**Objective:**

The present review collects the released articles about wearable devices or method for automatic caloric assessments.

**Method:**

A literature research has been performed with PubMed, Google Scholar, Scopus and ClinicalTrials.gov search engines, considering released articles regarding applications of wearable devices in eating environment, from 2005 onwards.

**Results:**

Several tools allow caloric assessment and food registration: wearable devices counting the number of bites ingested by the user, instruments detecting swallows and chewings, methods that analyse food with digital photography. All of them still require more validation and improvement.

**Conclusion:**

Automatic recording of caloric intake through wearable devices is a promising method to monitor body weight and eating habits in clinical and non-clinical settings, and the research is still going on.

## 1 INTRODUCTION

Nowadays, worldwide health care systems are focused on the creation of new management strategies to prevent worsening of chronic diseases and reduce associated economic costs. Non-communicable diseases (NCDs) are responsible for almost 38 million of deaths each year due to cardiovascular events, respiratory pathologies, cancer and diabetes. In high and middle income countries, NCDs are mostly related to unhealthy diets, sedentary lifestyle, tobacco smoke and alcohol abuse. Particularly, the excess of body weight, defined as a Body Mass Index higher than 25 kg/m^2^, the low consumption of fruits and vegetables and the consumption of foods with high content of fats and sugar, are main concerns of current health policies [1].

Data from observational studies reveal that the global phenomenon of obesity has nearly doubled between 1980 and 2008: percentage of obese men increased from 5% to 10%, while percentage of obese women increased from 8% to 14% [2]. According to the CUORE project, between 1998 and 2002, 17% of Italian men and 21% of Italian women were obese [3]. The World Health Organization (WHO) suggests the importance of healthy food choices to reduce consequences of food-related pathologies such as obesity, diabetes, cancer and cardiovascular diseases [4]. These recommendations are widely supported by scientific literature on dietary style: intake of healthy foods has positive effects on health conditions by reducing risks of recurrent coronary heart disease [5], positively modulating markers of inflammation in patients with diabetes [6] and improving health status of subjects with obstructive sleep apnea syndrome [7]. The maintenance of a positive energy balance is ensured by the complementary action of energy taken from nutrients (energy intake) and energy expended through physical activity and resting metabolic rate (energy expenditure) [8]. Several factors such as gender, age, environmental temperature, general health status and physical activity can influence energy intake and energy expenditure, suggesting the necessity of an energy balance calibrated for each subject [9].

As regards economic expenditure of obesity, an annual medical cost of 3613$ pro-capita has been estimated for women, and 1152$ pro-capita for men in United States [10]. A similar trend is observable in Italy, where costs related to health-care system are strongly influenced by clinical status of individuals: compared with normal-weighted subjects, annual pro-capita expenditure rises to 65€ in overweight subjects and to 105€ in obese subjects [3].

Because of the importance of preventing chronic diseases that may result from excess of body weight and unhealthy diet, clinical trials and epidemiological studies have developed different research instruments in order to collect reliable data on dietary patterns of large populations. Classic tools for food assessment include 24-hours dietary recall, dietary record, dietary history and food-frequency questionnaire. All these methods give subjective measure using a predefined or open-ended, self- or interviewer-administered format [11]. Despite several advantages, such as low cost, suitability and accuracy, food questionnaires are not free from potential errors: the inclusion of open-ended questions can be time-consuming, patients cannot be able to remind daily diet, and predefined answers can be inaccurate. Moreover, the long-term monitoring increases the risk of underestimating the energy intake and return of incorrect or incomplete information about the quality and type of nutrient intake [12].

For these reasons, the assessment of caloric intake and energy expenditure is a crucial challenge for the maintenance of individual and public health. An accurate monitor-activity is essential to obtain information about the eating habits of subjects at risk (e.g. obese and diabetic), subjects whose dietary assessment is difficult and often inaccurate (e.g. elderly and children) and also of healthy subjects. This is even more important considering that a diet-therapy requires long period of treatment and often involves subjects at home.

 A constant and accurate monitoring of calories and nutrient quality is essential both for the healthcare provider and the patient, to improve therapy effectiveness and compliance to diet. New wearable devices for caloric intake seem to be the future of scientific research on nutrition: easy to use, accurate, free of subjective bias, these new electronic tools are able to collect large amount of data.

These wearable devices can be divided into three macro categories according to the object or action captured by the device:

1. Devices capturing the gestures related to nutrition, such as arm and wrist movements;
2. Devices capturing sounds and vibrations from chewing and/or swallowing;
3. Devices that identify the kind and the amount of food analyzing food digital images.

The aim of the present paper is to review wearable devices or methods for automatic caloric assessment which are currently on a research and development process, reporting for each tool a brief description, a case study and some consideration about pros and cons.

## 2 MATERIALS AND METHODS

The literature search started from PubMed search engine, using the string: (“device”[Title] OR “bite”[Title] OR “armband”[Title]) AND (“caloric intake”[Title] OR “intake”[Title]). The results allowed the extraction of the names of the devices described in this review. These names were searched through Google Scholar, PubMed, Scopus and ClinicalTrials.gov search engines. Papers published from 2005 onwards and dealing with application of the cited wearable devices in eating environment were considered. Articles regarding the same devices in other applications (i.e. physical movement, hydraulic, veterinary) were excluded. No literature reviews about this topic were found. A flowchart of the papers’ selection process is represented in Fig. (**1**).

## 3 RESULTS

Results are shown in Table **1**, a list of each device/method with its description, experimental studies related to the specific device and method, and consideration about pros and cons. Four devices (Bite-counter, Autodietary, E-button, piezoelectric sensor-based necklace) and one method for caloric assessment which requires a personal smartphone (digital photography) are described. The devices or methods were tested in experimental studies to perform a process of validation, by asking participants to eat different kinds of food. The relative results provided an estimation of the accuracy of each tool, with consideration about limits and future developments.

## 4 DISCUSSION

Among devices containing inertial sensors (accelerometers and gyroscopes), capable of detecting the gesture of a body portion (arm or wrist) in a given space, the bite-counter measures both the vertical movement of the arm from the top downwards and the rotational movement of wrist. The gesture is translated into number of bites and these are translated into energy intake through the use of algorithms whose rational is based on the existence of a relationship between the number of bites and the number of calories eaten. In fact, although the instrument is not able to detect the type of food and the quality of the nutrients, and the calories per bite vary according to the food taken, the number of bites remains positively correlated to individually introduced calories [4].

Bite counter is one of the most promising self-monitoring tools for energy intake detection, both in healthy subjects and patients, thanks to its convenience and simplicity. Its use is already widespread and implemented in clinical and non-clinical setting [6, 11, 12]. Nevertheless, some limitations of the bite-counter must be acknowledged. First, bite-counter is susceptible to similar movements to the typical nutrition gestures and tends to register as bites movements of different nature. Second, it requires an active role of the subject in activating the tool and placing it on the dominant hand during the meal. Last, bite-counter does not provide any information about the quality of the food introduced [5, 6].

Given its high potential for use in health research, bite-counter needs some improvements for a more accurate and reliable detection of caloric intake.

The electronic devices that detect swallowing and/or chewing sounds are often very accurate in the identification of the type of food, although they are able to recognize only a limited number of foods [7] and do not provide information on the caloric intake. In more recent studies they are associated with other devices, such as motion and proximity sensors [8, 9], which are useful to reduce potential errors associated with movements of different nature and to obtain additional information. The biggest drawback of these tools is that they are uncomfortable to wear: in most cases, they must be worn as collars, and require careful use by the subject which has to minimize noise and accessory movements.

Finally, devices that capture digital images of foods before and after eating, allow the estimate of caloric intake from the analysis of pictures. Considering calories monitoring in free-living conditions, the Remote Food Photography Method **(**RFPM) is a semi-automatic method and its evolution is still in place: its function is based on the association of similar foods images by type and portion. Calculation software then allows the calculation of the calories and the analysis of the quality of nutrients introduced, providing a wide range of useful information for the subject and the researcher. The image technique takes advantage of common technologies, such as Smartphone, crossing data from multiple different database containing essential information on foods. However, detection is often affected by the quality of the image, by the position of food, and an important collaboration is requested from the subject.

## CONCLUSION

Self-monitoring of caloric intake becomes more urgent as wrong eating habits of developed countries increase. Moreover, health problems like obesity, diabetes, or other diseases with a high need for dietary assistance require instruments for a major control of caloric intake. New technologies based on wearable devices allow people to monitor their energy and caloric intake of their meals. Different methods are available, but all of them are still in progress. Wrist devices based on the arm movement and bite counting seem to be the most comfortable, cheap and easy to use: they still need a correlation between the number of bites and calories ingested. Collars with sensors for the detection of chewing and swallowing showed high accuracy, but they lack in comfort and still do not give information about caloric intake. Methods of food photography showed accuracy in caloric content of foods, and are very accessible with instruments of common use as smartphones, but they are strictly bounded to the user’s intervention and images qualities. For these reasons, further research is needed on this field.

## Figures and Tables

**Fig. (1):**
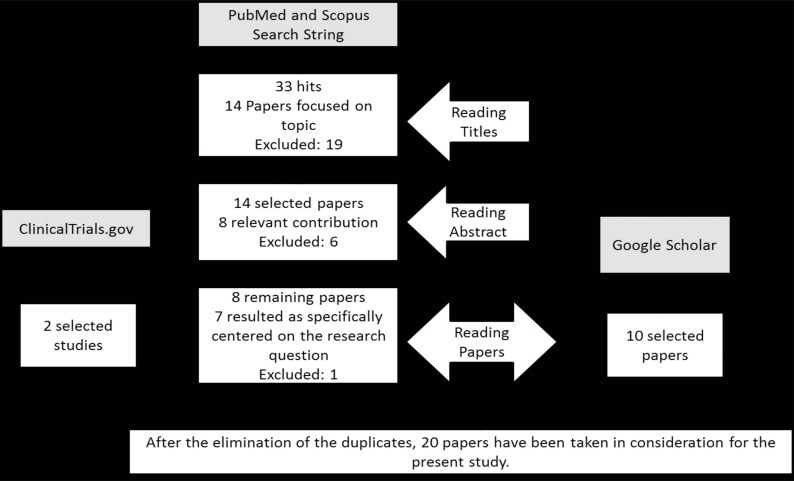
Flowchart of the review method.

**Table 1:** Devices description, experimental studies. Pros and cons.

**DEVICE DESCRIPTION**	**EXPERIMENTAL STUDIES**	**PROS AND CONS**
A **bite-counter** is a wearable device with an integrated tri-axial accelerometer and gyroscope. It appears as a clock and must be worn on the wrist, to record the number of bytes that the user ingests during a meal. This information is extracted from the analysis of wrist torsional movement, and the wrist movement occurring when the subject brings food from the plate to his mouth [6, 11, 12]. Starting from the number of bites recorded during a meal, an equation calculates the total calories ingested. An example of algorithm for the detection of the number of bites, coming from the data collected with an inertial sensor, was developed by Dong *et al* [10]. Salley **et al*.* developed a predictive equation to estimate the caloric amount associated with a single bite [6].	A study by Desedorf **et al*.* [5] assesses the effectiveness of the bite-counter in monitoring the caloric intake. 15 subjects between 23 and 58 years old were invited to eat different types of food and beverages, with different table utensils and in different ways. For each participant, the number of bites was detected both through the bite-counter and the visual monitoring of an observer. Findings reported in Table **2** show that the electronic tool tends to underestimate the actual number of bites: this happens especially when food or drink is consumed with a spoon, with a straw or with a fork. This is related to a reduced wrist rotation in some particular actions. For example, those subjects who eat soup with the spoon tend to stiffen the wrist while approaching food to the mouth, avoiding to spill the soup from the spoon. The bite-counter also requires an interval of at least 8 seconds between two consecutive bites: for a person who eats quickly, all the consecutive bites made in a shorter temporal interval are not detected. On the contrary, the bite-counter overestimates the number of bites when the meal is eaten with both fork and knife. In general, the difference between the manual measurement and the electronics one is reduced when food is eaten with hands, for both liquid and solid foods. The predictive equation developed by Salley **et al*.* [6]to determine the caloric amount estimation of a single bite, is based on individual and physical data, without taking into account the specific food. Multiple regression is based on height, weight, waist-to-hip ratio, gender and age of the subject. The authors validated and developed the model by collecting data from 280 participants: the results show that the estimate of caloric intake with the equation was more accurate than human-based estimation, with an average estimation error which went from -257.36 ± 790.22 kcal in the case of human estimation to 71.21 ± 562.14 kcal using the bites based method.	Thanks to its design and comfort, Bite-counter does not affect eating gestures and can be easily managed by the subjects. Nevertheless, as reported in some validation studies [4-6], both the algorithm for bite detection and the equation for caloric estimation lack in accuracy. Future research should focus on the development of an algorithm for bite counter which takes into account the numerous and complex gestures performed by the wrist while eating, and of a prediction equation for caloric estimation which includes both individual features and food type [4].
**AutoDietary** is an electronic device for food recognition, based on the distinction between chewing and swallowing sounds produced while eating. The system is composed by a necklace which records sounds of mastication through acoustic sensors, and a smartphone or tablet application which allows data transmission and elaboration, and gives the user an information about food type.	The study of Bi **et al*.* [13] aims to evaluate the efficacy of AutoDietary system in recognizing different types of food and drink. 12 participants were invited to eat seven kinds of food of different consistency. The experiment was performed in a laboratory with a low noise. The effectiveness of the instrument was assessed by considering the accuracy in the detection of the event, i.e. the sequence of chewing/swallowing sounds alternated with periods of silence, and the accuracy in the detection of the type of food, as represented in Table **3**.	AutoDietary is effective in food recognition, but it does not give information about caloric intake. Accuracy in food recognition is influenced also by the collar: it should fit snugly to the neck, otherwise the detection is incorrect or incomplete; for example, a participant with a low BMI reported a low percentage of accuracy. The effectiveness in recognizing the type of food also depends on the bite size: the smaller the food, the harder its recognition.
The rational of this device is the idea that foods can be identified by the energy exerted in their mastication: the microphone placed in correspondence of the neck, close to the jaw, converts superficial skin vibrations into acoustic signals. Following the algorithm used by Bi **et al*.* [13], this signal is analysed through the distinction between chewing and swallowing events, and the extraction of more than 30 features representing its acoustic characteristics. An algorithm based on a light-weight decision tree is used to recognize the type of food intake.	The results show that AutoDietary has a high performance in food recognition, especially in distinguishing solid from liquid food. Fontana *et al* study [9]. tested the effectiveness of an energy intake detection model based on Counts of Chews and Swallows (CCS). For the creation of CCS model, numbers of chews and swallows were extracted for each subject using a throat microphone, in order to estimate the average mass of liquid and solid food ingested per chewing/swallowing: these parameters were used to create predictive models to estimate the intake of solid and liquid food. The model was then validated in a second step, by verifying the ability of the method to generalize the predictive estimates in different foods than those used for the initial test. The energy content of each food and caloric intake per meal was calculated by multiplying the estimated mass of food, obtained from the count of chews and swallows, by the caloric density extracted from nutritional analysis. The results show that the method still commits some errors, but they might be compensated adding correction factors in the model. Nevertheless, this study suggests a promising alternative to diet diaries.	The results from questionnaires performed by the participants in the study of Bi **et al*.* [13], determined that the device appeared acceptable for most users, in terms of comfort and functionality. The instrument was tested (and it is used) only for a limited number of foods. For these reasons, the developers planned to further improve the device, by performing experimental tests in a real-life environment, considering a larger number of foods, and enhancing device capabilities adding the chance to perform an estimate of caloric intake by detecting the volume and weight of food ingested.
The **piezoelectric sensor-based necklace** records the mechanical stress caused by the swallowing action. It detects skin vibrations in the low part of trachea at the moment of food passage. Data from the sensor are then sent via Bluetooth to a smartphone app. There are two versions of the collar. “Sportband style collar” sacrifices appearance for a higher sensor stability: because of his major adherence to the neck, it prevents lateral motions which can bring to false positives. For these reasons, this kind of instrument is more indicated for clinical and scientific environment. Otherwise, a more comfortable “pendant-based” version is available, with a lower neck adherence but a higher sensitivity to movements not related to eating [8]. The device is able to recognize the ingestion of solid and liquid foods by detecting the movements occurring between two swallows: a swallowing preceded by numerous chewing movements indicates the assumption of a solid food; a series of consecutive swallows without movements indicates the assumption of liquid foods. The algorithm for food recognition developed by Kalantarian **et al*.* [8] performed swallow identification through the analysis of the voltage signal coming from the piezoelectric sensor. To perform activity recognition, the authors added a tri-axial accelerometer to the necklace, in order to detect movements of the upper body which might be mistaken as swallows.	Kalantaria **et al*.* [8] performed an experimental study on the effectiveness of the collar with built-in piezoelectric sensor and accelerometer. 30 subjects between 22 and 34 years old were recruited: all participants were invited to eat foods with different textures. All subjects wore the sportband collar with accelerometer. Results reported in Table **4** show the accuracy in the type of food recognition, where accuracy was defined as the percentage of swallows correctly identified. A strong presence of false positives was observed when the instrument was not associated to the accelerometer: in this case, the percentage of movements incorrectly classified as swallowing increases to 18% with a head rotation to the left, 46% with a head rotation upward and 8% during a walk motion.	The piezoelectric-sensor based necklace is a low-cost device for food recognition. Thanks to the interface with the smartphone app, it can easily provide the user information about food consumption, but it does not give information about the caloric intake. Results showed that the tight configuration is too uncomfortable to be worn for more than a few minutes. Tightening the necklace, the movements of the piezoelectric sensor are restricted thus decreasing the sensitivity of detection. On the contrary, the loose configuration causes too much fluctuations in the signal, making the results unusable. For these reasons, a compromise between comfort and accuracy must be found: the use of an accelerometer might help this way.
**E-Button** is a wearable computer collecting and processing data about physical activity and diet. Because of its small dimensions and weight, it can be worn on the chest. It contains two wide-angle cameras with stereo and depth information, an UV sensor which distinguishes indoor and outdoor places, an IMU (Inertial Measurement Unit) composed by a 3-axis accelerometer, a 3-axis gyroscope and a magnetometer, an audio processor, a proximity sensor to record arm/hand motion in front of the chest, a barometer measuring the distance from the device to the floor, and a GPS. It can communicate wireless via Wi-Fi or Bluetooth with a smartphone or tablet. Considering its use as a dietary device, e-Button camera collects images with a determined rate. These pictures are analysed to determine the type of food that the user is eating, through techniques of computer vision. First, shapes of utensil are detected, then food regions are segmented based on colour, texture and a complexity measure. Next, the volume of each dish is determined, with the comparison of food-specific shape models. After food and volume detecting, calories information is extracted from a database like the Food and Nutrient Database for Dietary Studies (FNDDS) [14].	The study proposed by Jia **et al*.* [15] aims to evaluate the accuracy of the electronic instrument e-button in determining the volume of food. 7 participants were involved in the study. The volume of each food was measured with a validated method (seed displacement method). Two different detection methods of food volume were compared, considering the same images detected by the device-button: the computer-based estimation, and a visual estimation performed by some raters by observing the same images analysed by the software. Considering the mean difference from the actual volume (measured with seed displacement), it resulted as -5% (SD of 21%) in the automatic detection, and -15.5% (SD 41.4%) in the visual detection. The results show that the volume of food can be automatically calculated from an image with a higher level of accuracy than a visual estimation. The E-button then provides an objective and accurate measurement of food intake.	E-Button allows the semi-automatic calculation of the food volume and does not require an excessive effort by the user. Moreover, its dimensions and position on the body, allow it to be easily worn in different situation without affecting users‘ habits: for this reason, it can be used in different assets, such as physical activity, sedentary behaviours and support for blind people. Nevertheless, some issues are still in progress. The device does not correctly identify all types of food, as beverages, condiments, not visible ingredients or cooking methods. The images ‘ quality depends on lighting in the room, the angle of the camera and the presence of foods with complex or confusingly shapes [15]. Finally, the high level of technology contained in the e-Button creates a significant economic cost if compared to other devices for food recognition.
**Digital photography of foods method** was used in cafeteria settings to perform the analysis of nutrients and daily calories assumed by the clients. According to this method, digital video cameras placed in the environment capture images of people food’s selection and food remaining on the plates (leftovers). Images of weighted standard portions are also collected, and associated to the information collected in the Food and Nutrient Database for Dietary Studies (FNDDS), providing nutrients and calories intake [16, 17]. The **Remote Food Photography Method (RFFM)** uses digital photography in free-living situations: it is a semi-automatic system in which the subject captures a picture of his dishes with his smartphone, before and after the meal. These images are sent in real-time through a wireless network to the Food Photography Application©, to be compared to other images with dishes of equivalent portions and same type of food contained in a database. The information about energy and nutrition intake are extracted from FNDDS [16].	Martin **et al*.* conducted a study to assess the reliability of RFPM in the estimation of daily caloric intake comparing the meals consumed in a laboratory settings and the meal consumed in an uncontrolled environment [18]. 50 participants were instructed to photograph the food with their smartphone and send the image to the Food Photography Application©, which returns information about the caloric intake and nutrients. Even in this case, the analysis of the food was performed by comparing the photographs taken by the participants with the images catalogued in a central archive. The archive consists of an ordered collection of thousands of types of foods associated with all the information necessary to estimate the caloric quantity, namely: • the size of a standard portion, useful for the estimation of the size of the actual portion of food; • the quality of the nutrients, available through the information gathered in the FNDDS. Results reveal that caloric intake estimation through RFPM in laboratory meals does not significantly underestimate the actual amount of energy (-5.5%), while for meals consumed in uncontrolled environment, the level of estimation decreases further becoming significant (-6.6%). Despite the tendency to underestimate the actual values, the RFPM method tends to underestimate an average caloric quantity of 97 kcal per day: however, that is a lower error if compared to other detection methods [16].	Digital photography for food method presents similar variability if compared to visual estimation [17]. Nevertheless, the method is still not applicable in free living conditions. Studies on RFPM [18, 19] showed that the method is accurate in caloric intake estimation in people’s natural environment. It was tested on various types of samples, including a population of mothers for the daily caloric intake of their child [20]. The method is strictly connected to the user’s intervention: individuals may forget to take pictures of foods or they can lose the Smartphone device. For these reasons, alarm/warning systems are designed [18]. Moreover, digital images must be of quality to be readable so the subjects must be instructed beforehand. Finally, certain types of food, such as seasonings, are difficult to analyse and often do not allow the precise energy intake calculation.

**Table 2:** Accuracy of the Bite counter in bite detection [5].

**Food (utensil)**	**Accuracy**
Meat (fork and knife)	127%
Sides (fork)	82.6%
Soup (spoon)	60.2%
Pizza (hands)	87.3%
Soda (hands)	81.7%
Smoothie (straw)	57.7%
Total	81.2%

**Table 3:** Accuracy of Autodietary in food recognition [13].

**Event/food**	**Accuracy**
Chewing-swallowing	86.6%
Apple	86.3%
Carrot	84.9%
Biscuit	82.9%
Chips	87.7%
Walnuts	75.5%
Peanuts	83.4%
Water	93.3%

**Table 4:** Accuracy of piezoelectric sensor-based necklace with accelerometer in food recognition [8].

**Food**	**Accuracy**
Water	81.4%
sandwich	84.5%
Chips	85.3%

## LIST OF ABBREVIATIONS

(NCDs): = Non-communicable diseases
(WHO): = World Health Organization
